# Juvenile idiopathic arthritis patients with positive family history of autoimmune thyroid disease might benefit from serological screening: analysis of the international Pharmachild registry

**DOI:** 10.1186/s12969-023-00802-1

**Published:** 2023-02-21

**Authors:** Joeri W. van Straalen, Laurie Baas, Gabriella Giancane, Lyudmila Grebenkina, Jurgen Brunner, Gabriel Vega-Cornejo, Vyacheslav G. Chasnyk, Liora Harel, Simone Appenzeller, Elisabeth Gervais, Sytze de Roock, Nico M. Wulffraat, Nicolino Ruperto, Joost F. Swart, for the Paediatric Rheumatology International Trials Organisation (PRINTO)

**Affiliations:** 1 Department of Pediatric Immunology and Rheumatology, Wilhelmina Children’s Hospital, University Medical Center Utrecht, P.O. box 85090, 3508 AB Utrecht, The Netherlands; 2 Faculty of Medicine, Utrecht University, Utrecht, the Netherlands; 3 Clinica Pediatrica e Reumatologia, IRCCS Istituto Giannina Gaslini, Genoa, Italy; 4 Dipartimento di Neuroscienze, Riabilitazione, Oftalmologia, Genetica e Scienze Materno-Infantili (DiNOGMI), Università degli Studi di Genova, Genoa, Italy; 5 Pediatric Department, Togliatti City Clinical Hospital №5, Togliatti, Russia; 6 Pediatric Rheumatology, Department of Pediatrics, Medical University Innsbruck, Innsbruck, Austria; 7 Danube Private University, Krems, Austria; 8 Clínica Pediátrica de Reumatología y Enfermedades Autoinmunes (CREA), Hospital México Americano, Guadalajara, Mexico; 9 Department of Hospital Pediatrics, Saint Petersburg State Pediatric Medical University, Saint Petersburg, Russia; 10 Pediatric Rheumatology Unit, Schneider Children’s Medical Center, Petach-Tikvah, Israel; 11 Sackler School of Medicine, Tel Aviv University, Tel Aviv, Israel; 12 Department of Orthopedics, Rheumatology and Traumatology, School of Medical Science, University of Campinas, Campinas, Brazil; 13 Rheumatology, Centre Hospitalier Universitaire (CHU) de Poitiers, Poitiers, France; 14 UOSID Centro trial, IRCCS Istituto Giannina Gaslini, Genoa, Italy

**Keywords:** Juvenile idiopathic arthritis, Autoimmune thyroid disease, Hashimoto’s disease, Graves’ disease, Screening, Epidemiology, Registry

## Abstract

**Background:**

Little is known about the association between juvenile idiopathic arthritis (JIA) and autoimmune thyroid disease (AITD) and therefore there are no indications for AITD screening in this population, which is possible using standard blood tests. The objective of this study is to determine the prevalence and predictors of symptomatic AITD in JIA patients from the international Pharmachild registry.

**Methods:**

Occurrence of AITD was determined from adverse event forms and comorbidity reports. Associated factors and independent predictors for AITD were determined using univariable and multivariable logistic regression analyses.

**Results:**

The prevalence of AITD after a median observation period of 5.5 years was 1.1% (96/8965 patients). Patients who developed AITD were more often female (83.3% vs. 68.0%), RF positive (10.0% vs. 4.3%) and ANA positive (55.7% vs. 41.5%) than patients who did not. AITD patients were furthermore older at JIA onset (median 7.8 years vs. 5.3 years) and had more often polyarthritis (40.6% vs. 30.4%) and a family history of AITD (27.5% vs. 4.8%) compared to non-AITD patients. A family history of AITD (OR = 6.8, 95% CI: 4.1 – 11.1), female sex (OR = 2.2, 95% CI: 1.3 – 4.3), ANA positivity (OR = 2.0, 95% CI: 1.3 – 3.2) and older age at JIA onset (OR = 1.1, 95% CI: 1.1 – 1.2) were independent predictors of AITD on multivariable analysis. Based on our data, 16 female ANA positive JIA patients with a family history of AITD would have to be screened during ±5.5 years using standard blood tests to detect one case of AITD.

**Conclusions:**

This is the first study to report independent predictor variables for symptomatic AITD in JIA. Female ANA positive JIA patients with positive family history are at increased risk of developing AITD and thus might benefit from yearly serological screening.

## Background

Juvenile idiopathic arthritis (JIA) is a diagnosis of exclusion that includes all forms of chronic arthritis of unknown origin with onset below the age of 16 years [1]. It is the most common childhood rheumatic disease with an estimated global incidence of 1.6 – 23 cases per 100,000 children [2] and often persists into adulthood [3]. The International League of Associations for Rheumatology (ILAR) distinguishes seven JIA categories with different clinical and laboratory measures [4], although another classification system is under development [5].

There is some evidence that children with JIA suffer more often from autoimmune thyroid disease (AITD) than the general pediatric population [6–8]. AITD comprises Hashimoto’s thyroiditis which causes hypothyroidism and Graves’ disease which causes hyperthyroidism. If undiagnosed and thus left untreated, hyper- and hypothyroidism may lead to a variety of complaints, such as constipation or diarrhea, irritability, fatigue, hair loss but ultimately also growth retardation and depression [9].

Currently, little is known about the association between JIA and AITD and therefore there are no indications for AITD screening in this population, which is possible using standard blood tests. Previous studies reported a prevalence of (subclinical) AITD in JIA varying from 1 to 44% [7, 8, 10–16]. One study of 81 JIA patients reported a significant association between a family history of thyroid disease and AITD [17]. Nevertheless, studies that primarily focus on AITD in JIA are scarce and most include not only symptomatic but also subclinical AITD. Furthermore, no study has yet established independent predictor variables for AITD in JIA.

The purpose of this study is to determine the prevalence of symptomatic AITD in JIA and moreover to identify independent predictors for AITD using data from the international observational Pharmachild registry.

## Methods

### Pharmachild

Pharmachild was set up in 2011 with the primary aim of studying safety and effectiveness of drug therapies in JIA. Pharmachild collects demographic, clinical and laboratory data of JIA patients from 85 Paediatric Rheumatology International Trials Organisation (PRINTO) medical centers from 31 countries across the globe [18]. Inclusion criteria are JIA classified according to ILAR criteria while under treatment or previously treated with nonsteroidal anti-inflammatory drugs (NSAIDs), intra-articular corticosteroids, systemic corticosteroids, and/or conventional synthetic (cs-) or biological (b-) disease-modifying antirheumatic drugs (DMARD) as per physician decision. The registry consists of two cohorts. The first is a cohort of all included patients with retrospective information about drug exposure and adverse events (AEs) from disease onset until registration into Pharmachild. The second is a cohort of patients with additional prospective information about disease activity and patient-reported outcomes for hospital visits after registration into Pharmachild. More information about the Pharmachild registry is published elsewhere [19]. Data lock occurred on 18 December, 2019 and all patients at that time were included in the present study.

### Outcome and determinants

The outcome of interest in this study was the ever occurrence of symptomatic AITD (Hashimoto’s thyroiditis, Graves’ disease and non-specified AITD). This outcome (yes/no) was evaluated for all patients from two sources: free-text fields for comorbidity reporting at registration into Pharmachild and AE forms. AEs in Pharmachild are reported using the Medical Dictionary of Regulatory Activities (MedDRA) coding system (version 22) with a three-level monitoring check for consistency by the treating physician, medical monitor (JS) and PRINTO certified MedDRA coders [20]. The following MedDRA preferred terms were considered as AITD: “hypothyroidism”, “autoimmune thyroiditis”, “thyroiditis”, “hyperthyroidism” and “Basedow’s disease”. Goiter and congenital thyroid disorder were not considered as AITD. Laboratory results were not considered for determining AITD, since these were likely to involve subclinical cases. All mentions of possible AITD cases were retrieved and reviewed by one researcher (LB) and event descriptions were subsequently independently evaluated by two other researchers (JS and JvS). In order to explore the coexistence of endocrinopathies, mentions of growth retardation/short stature, diabetes mellitus and celiac disease were retrieved from both free-text comorbidity reports and AE forms. For this assessment, the following mentions were included: “growth retardation”, “growth retarded”, “short stature”, “stature short”, “coeliac disease”, “celiac disease”, “type 1 diabetes mellitus”, “diabetes”, “type I diabetes mellitus” and “diabetes mellitus insulin-dependent”. In addition, the following patient characteristics were collected for all patients: sex, ethnicity, age at JIA onset, ILAR category, anti-nuclear antibodies (ANA) status, rheumatoid factor (RF) status, human leukocyte antigen (HLA)-B27 status, family history of autoimmune disease and AITD (in first, second and/or third-degree relatives), observation period (time from JIA onset until last Pharmachild visit), the number of active joints at JIA diagnosis (max. 12 months later) and drug history at last visit. Ethnicity was reported by the treating physician from a fixed set of categories. For ANA positivity, only one positive ANA test was required. A positive RF status was defined as two positive RF determinations at least 3 months apart. Drugs included were NSAIDs, intraarticular corticosteroids, systemic corticosteroids, cs- and b-DMARDs.

### Statistical analysis

The retrospective and prospective Pharmachild cohorts were analyzed together. Patient characteristics were compared between patients with and without AITD using univariable logistic regression analyses. When the 95% confidence interval (CI) of the odds ratio (OR) did not contain 1, this was considered a statistically significant effect. All variables that differed statistically significant between AITD and non-AITD patients were considered for inclusion into a complete case multivariable logistic regression model in order to identify independent predictors of AITD. Predictors were selected using a stepwise backward procedure based on the Akaike’s Information Criterion (AIC). This measure is used to select a model that best predicts the observed data while adding a penalty for the number of variables in the model [21]. Because onset dates of AITD were not available for all cases, the observation period and drug history were not considered for inclusion into the multivariable model. The active joint count was also not considered since this measure was only available for (part of the) patients from the prospective Pharmachild cohort. Numerical variables were tested for a linear relationship with the logit outcome using the Box-Tidwell test. The performance of the multivariable model in distinguishing between AITD and non-AITD patients was evaluated by the area under the receiver operating characteristic curve (AUC). Based on the prevalence of AITD in patients at increased risk for developing AITD following our prediction model, we calculated a number needed to screen (NNS) (1 divided by the absolute risk reduction). IBM SPSS statistics (version 25.0.0.2) and R (version 4.0.3) were used for the statistical analyses.

## Results

### Patient characteristics

**Fig. 1:**
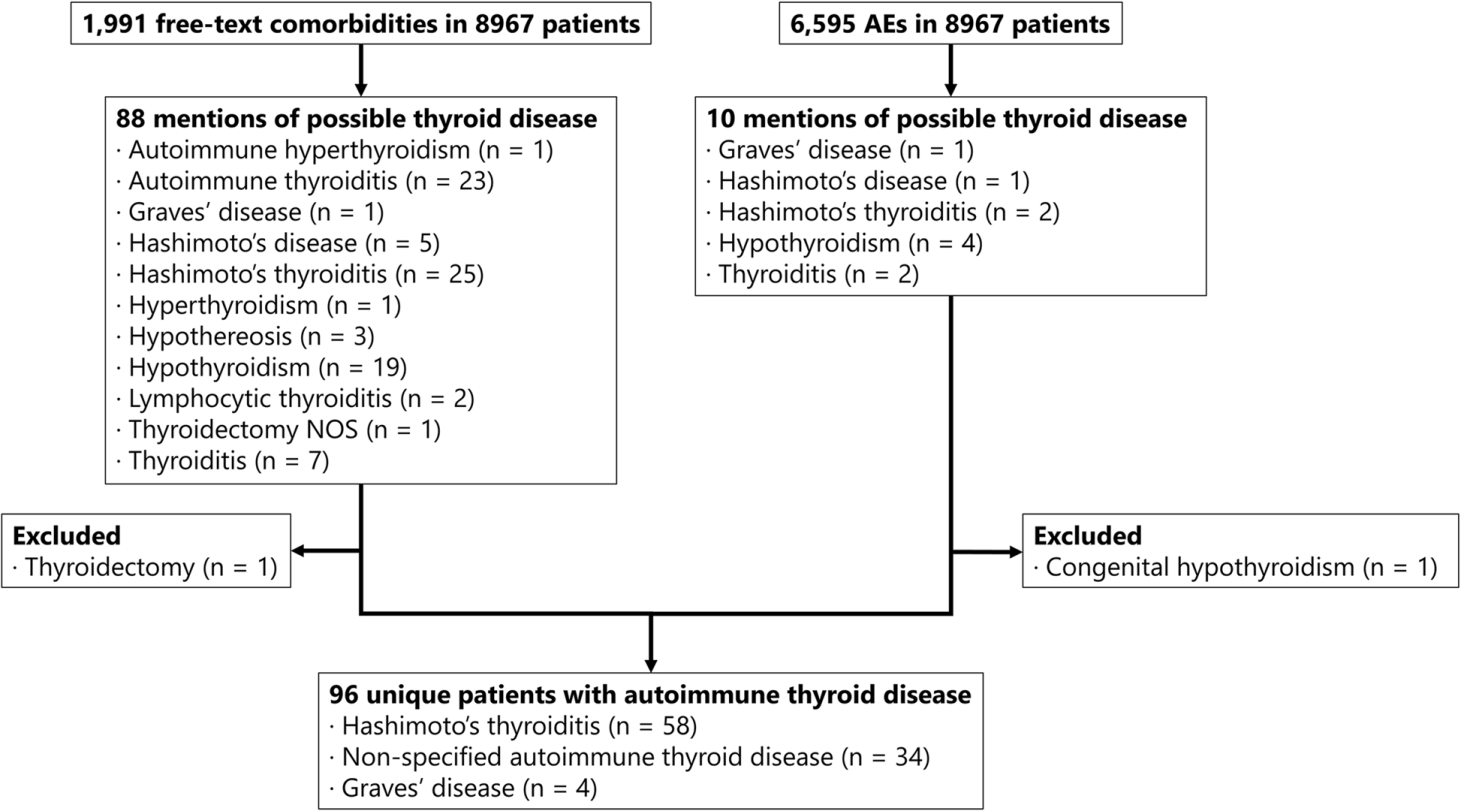
Flowchart of selected AITD cases. AE: adverse event, NOS: not otherwise specified

A total of 8965 patients were included from the Pharmachild registry for analysis with a total observation period of 57,053 years (median 5.5 years, IQR: 2.8–9.0). Within these patients, 96 cases of clinical AITD (1.1%) were identified (Fig. 1). Hashimoto’s thyroiditis occurred in 58/96 (60.4%) cases and Graves’ disease in 4/96 (4.2%) of cases. The remaining 34/96 (35.4%) of AITD cases were unspecified. Patients who developed AITD were more often female, RF positive and ANA positive than patients who did not develop AITD (Table 1). Furthermore, AITD patients were older at JIA onset and had more often polyarthritis and a family history of autoimmune disease and AITD compared to non-AITD patients. Celiac disease and diabetes mellitus were reported significantly more often in AITD patients compared to patients without AITD: 4.2% vs. 0.6% (*P* < 0.01) and 2.1% vs. 0.3% (*P* = 0.04), respectively. No significant difference was found for growth retardation/short stature: 0.0% for AITD patients and 0.3% for non-AITD patients (*P* = 1.0). AITD patients had less often systemic arthritis than non-AITD patients. The observation period, HLA-B27 status, active joint count at JIA diagnosis, ethnicity and drug history at last visit did not differ significantly between the two groups. A distribution of different AITD cases per ILAR category is provided in Table 2.

**Table 1:** Characteristics of patients in Pharmachild with and without AITD

	Total (*n* = 8965)	No AITD (*n* = 8869)	AITD (*n* = 96)
Observation period in years, median (IQR)	5.5 (2.8 – 9.0)	5.5 (2.8 – 9.0)	4.9 (3.0 – 9.5)
Female sex, n (%)	6107 (68.1%)	6027 (68.0%)	80 (83.3%)
Ethnicity, n (%)			
*European*	6940 (86.9%)	6853 (86.9%)	87 (90.6%)
*Hispanic*	249 (3.1%)	248 (3.1%)	1 (1.0%)
*Indian*	145 (1.8%)	144 (1.8%)	1 (1.0%)
*Middle Eastern*	196 (2.5%)	193 (2.4%)	3 (3.1%)
*Multiethnic*	110 (1.4%)	109 (1.4%)	1 (1.0%)
*North African*	154 (1.9%)	152 (1.9%)	2 (2.1%)
*Southeast Asian*	64 (0.8%)	64 (0.8%)	0 (0.0%)
*Sub-Saharan African*	78 (1.0%)	78 (1.0%)	0 (0.0%)
*Other*	48 (0.6%) *n* = 7984	47 (0.6%) *n* = 7888	1 (1.0%) *n* = 96
Family history of autoimmune disease, n (%)	2632 (30.4%) *n* = 8669	2584 (30.1%) *n* = 8578	48 (52.7%) *n* = 91
Family history of AITD, n (%)	441 (5.1%) *n* = 8669	416 (4.8%) *n* = 8578	25 (27.5%) *n* = 91
Age at JIA onset in years, median (IQR)	5.3 (2.4 – 9.9)	5.3 (2.4 – 9.9)	7.8 (3.1 – 12.7)
ILAR category, n (%)			
*Enthesitis-related arthritis*	969 (10.8%)	961 (10.8%)	8 (8.3%)
*Oligoarthritis*	3370 (37.6%)	3338 (37.6%)	32 (33.3%)
*Polyarthritis (RF-)*	2371 (26.4%)	2341 (26.4%)	30 (31.3%)
*Polyarthritis (RF+)*	367 (4.1%)	358 (4.0%)	9 (9.4%)
*Psoriatic arthritis*	298 (3.3%)	292 (3.3%)	6 (6.2%)
*Systemic arthritis*	968 (10.8%)	966 (10.9%)	2 (2.1%)
*Undifferentiated arthritis*	622 (6.9%)	613 (6.9%)	9 (9.4%)
Active joint count at JIA diagnosis, median (IQR)	2.0 (0.0 – 5.0) *n* = 666	2.0 (0.0 – 5.0) *n* = 660	3.0 (1.3 – 4.0) *n* = 6
ANA positive, n (%)	3486 (41.7%) *n* = 8365	3437 (41.5%) *n* = 8277	49 (55.7%) *n* = 88
RF positive, n (%)	342 (4.3%) *n* = 7876	333 (4.3%) *n* = 7786	9 (10.0%) *n* = 90
HLA-B27 positive, n (%)	1122 (20.7%) *n* = 5414	1114 (20.8%) *n* = 5363	8 (15.7%) *n* = 51
Drug history at last visit, n (%)			
*NSAIDs*	7375 (82.3%)	7298 (82.3%)	77 (80.2%)
*Intraarticular corticosteroids*	4545 (50.7%)	4496 (50.7%)	49 (51.0%)
*Systemic corticosteroids*	3582 (40.0%)	3551 (40.0%)	31 (32.3%)
*cs-DMARDs*	7795 (86.9%)	7712 (87.0%)	83 (86.5%)
*Methotrexate*	7524 (83.9%)	7446 (84.0%)	78 (81.2%)
*b-DMARDs*	5946 (66.3%)	5878 (66.3%)	68 (70.8%)
*Anti-TNF*	5248 (58.5%)	5183 (58.4%)	65 (67.7%)

*AITD* autoimmune thyroid disease, *ANA* anti-nuclear antibodies, *b* biological, *cs* conventional synthetic, *DMARDs* disease-modifying antirheumatic drugs, *HLA* human leukocyte antigen, *ILAR* International League of Associations for Rheumatology, *JIA* juvenile idiopathic arthritis, *NSAIDs* nonsteroidal anti-inflammatory drugs, *RF* rheumatoid factor, *TNF* tumour necrosis factor

**Table 2:** Distribution of different AITD cases per ILAR category

ILAR category	Total AITD (*n* = 96)	Hashimoto’s disease (*n* = 58)	Graves’ disease (= 4)	Unspecified AITD (*n* = 34)
Enthesitis-related arthritis	8 (8.3%)	6 (10.3%)	0 (0.0%)	2 (5.9%)
Oligoarthritis	32 (33.3%)	17 (29.3%)	3 (75.0%)	12 (35.3%)
Polyarthritis (RF-)	30 (31.3%)	21 (36.2%)	0 (0.0%)	9 (26.5%)
Polyarthritis (RF+)	9 (9.4%)	4 (6.9%)	0 (0.0%)	5 (14.7%)
Psoriatic arthritis	6 (6.2%)	3 (5.2%)	1 (25.0%)	2 (5.9%)
Systemic arthritis	2 (2.1%)	1 (1.7%)	0 (0.0%)	1 (2.9%)
Undifferentiated arthritis	9 (9.4%)	6 (10.3%)	0 (0.0%)	3 (8.8%)

*AITD* autoimmune thyroid disease, *ILAR* International League of Associations for Rheumatology, *RF* rheumatoid factor

### Predictors for AITD

On multivariable analysis, a family history of AITD, female sex, ANA positivity and older age at JIA onset were independent predictors of AITD (Table 3). This model included 7345 patients and 82 AITD events due to 1620 patients with missing data. The model had good discriminatory power (AUC = 0.71, 95% CI: 0.65 – 0.78). Based on the data in Pharmachild, the number of female ANA positive JIA patients with a family history of AITD needed to screen to detect one case of AITD is 16. This number decreases with increasing age at JIA onset (Table 4).

**Table 3:** Associated factors and independent predictors for AITD on univariable and multivariable analysis

	Univariable analysis		Multivariable analysis	
Variable	OR	95% CI	OR	95% CI
Family history of AITD	7.43	4.56 – 11.74^c^	6.84	4.07 – 11.14^c^
Female sex	2.36	1.42 – 4.19^c^	2.22	1.25 – 4.25^c^
ANA positive	1.77	1.16 – 2.71^c^	1.99	1.25 – 3.18^c^
Age at JIA onset in years	1.10	1.05 – 1.15^c^	1.12	1.07 – 1.18^c^
Observation period in years^a^	1.02	0.98 – 1.07		
Ethnicity				
*European*	1.00	Reference		
*Sub-Saharan African*	–	–		
*Hispanic*	0.32	0.02 – 1.44		
*Indian*	0.55	0.03 – 2.48		
*Middle Eastern*	1.22	0.30 – 3.30		
*Multiethnic*	0.72	0.04 – 3.29		
*North African*	1.04	0.17 – 3.32		
*Southeast Asian*	–	–		
*Other*	1.68	0.09 – 7.80		
Family history of autoimmune disease	2.59	1.71 – 3.93^c^		
ILAR category				
*Oligoarthritis*	1.00	Reference		
*Enthesitis-related arthritis*	0.87	0.37 – 1.80		
*Polyarthritis (RF-)*	1.34	0.81 – 2.21		
*Polyarthritis (RF+)*	2.62	1.17 – 5.31^c^		
*Psoriatic arthritis*	2.14	0.80 – 4.81		
*Systemic arthritis*	0.22	0.03 – 0.71^c^		
*Undifferentiated arthritis*	1.53	0.68 – 3.09		
Active joint count at diagnosis^b^	0.99	0.82 – 1.07		
RF positive	2.49	1.15 – 4.73^c^		
HLA-B27 positive	0.71	0.31 – 1.43		
Drug history at last visit^a^				
*NSAIDs*	0.87	0.54 – 1.49		
*Intraarticular corticosteroids*	1.01	0.68 – 1.52		
*Systemic corticosteroids*	0.71	0.46 – 1.09		
*cs-DMARDs*	0.96	0.55 – 1.81		
*Methotrexate*	0.83	0.51 – 1.43		
*b-DMARDs*	1.24	0.80 – 1.95		
*Anti-TNF*	1.49	0.98 – 2.32		

*AITD* autoimmune thyroid disease, *ANA* anti-nuclear antibodies, *b* biological, *CI* confidence interval, *cs* conventional synthetic, *DMARD* disease-modifying antirheumatic drug, *HLA* human leukocyte antigen, *ILAR* International League of Associations for Rheumatology, *JIA* juvenile idiopathic arthritis, *NSAIDs* nonsteroidal anti-inflammatory drugs, *OR* odds ratio, *RF* rheumatoid factor, *TNF* tumor necrosis factor

^a^Not considered for multivariable analysis due to missing AITD onset dates

^b^Only available for patients from the prospective cohort and therefore not considered for multivariable analysis

^c^statistically significant effect

**Table 4:** Number of high-risk JIA patients needed to screen (NNS) to detect a case of AITD. The table summarizes the number of ANA positive girls with a family history of AITD who would have to be screened during a median observation period of 5.5 years to detect one case of AITD as a function of the age at JIA onset

Age at JIA onset (years)	AITD prevalence	NNS
≥0	14/196 (7.1%)	16
≥4	10/85 (11.8%)	9
≥8	5/34 (14.7%)	7
≥12	4/18 (22.2%)	5

*AITD* autoimmune thyroid disease, *ANA* anti-nuclear antibodies, *JIA* juvenile idiopathic arthritis, *NNS* number needed to screen

## Discussion

The prevalence of AITD observed in the current study (1.1%) was lower compared to prevalence rates reported in the majority of previous studies about AITD in JIA (5.0 – 44.4%) [7, 8, 10, 11, 14–17]. These studies, however, also included cases of subclinical AITD based on active screening for serum levels of thyroid hormones (T_3_ and T_4_), thyroid-stimulating hormone (TSH) and anti-thyroid antibodies (TgA: thyroglobulin antibodies and/or TPOA: thyroid peroxidase antibodies). Two previous studies focused on clinical AITD in JIA and found similar prevalence rates as the current study (0.8 and 1.3%) [12, 13]. AITD has a varying prevalence in the general pediatric population (0.1 – 9.6%) according to the criteria used for diagnosis [22–26]. A population-based study from Scotland focused on clinical hypothyroidism in young people aged < 22 years and found a prevalence of 0.14% [27], which is over 4 times as low as the prevalence of clinical hypothyroidism in JIA found in the current study. In addition, several studies reported increased serum levels of anti-thyroid antibodies in children with JIA compared to healthy controls [7, 8, 15, 17].

Our study highlighted as independent predictors of AITD in JIA a family history of AITD, female sex, a positive ANA status and older age at JIA onset. In fact, previous studies about AITD in JIA also report a female predominance in the AITD group [7, 8, 10, 11, 17]. This can be explained by the predominance of girls in most JIA categories [28] and autoimmunity in general [29]. Previous studies have suggested that oligoarthritis might be associated with AITD in JIA [7, 8, 11, 15], but with the current study we conclude that this effect is likely explained by ANA positivity and female sex, which is highly frequent in oligoarthritis [28]. Similarly, the association between AITD and RF positivity and RF+ polyarthritis that we observed in univariable analyses is probably explained by older age and female sex [28].

This is the first study to report an (adjusted) association between AITD and a positive ANA status in JIA, likely due to a limited sample size in previous studies. An association between thyroid disorders and ANA positivity has previously been reported in adult RA [30] and a raised prevalence of ANA in AITD patients has also been previously reported, although the mechanism behind this phenomenon is not known [31–33]. More interestingly, we found a significant association between AITD in JIA patients and a family history of AITD, as described before in another study [17]. Previous studies on AITD patients have also reported high frequencies of familial AITD [34–36] or familial autoimmune disease in general [37]. The association between older age at JIA onset and AITD has not been previously reported. We hypothesize that this effect is caused by merely age rather than age at JIA onset, since older patients in general have an increased cumulative risk of developing any disease including AITD. In fact, it is known that the prevalence of pediatric AITD peaks during adolescence [25, 26, 38]. Systemic arthritis was observed considerably less in AITD patients than non-AITD patients in the current study, which might be explained by the fact that this JIA category resembles more an auto-inflammatory rather than an autoimmune disease and does not predominantly affect girls [39].

After a median observation period of 5.5 years, we observed a considerable increase in AITD prevalence for JIA patients at increased risk for developing AITD according to our analyses, providing rationale for yearly AITD screening in this high-risk group. Based on the Pharmachild data, only five ANA positive girls with a family history of AITD and an age at JIA onset of ≥12 years would have to be screened during 5.5 years to detect one case of clinical AITD. According to the coefficients in our prediction model, it is safe to conclude that a family history of AITD is the most important predictor of AITD in JIA, with an even larger OR for AITD than a 10-year increase in age at JIA onset. Hence, this would be the most important factor for clinicians to determine in JIA patients when estimating the risk of developing AITD. Screening for thyroid disease is based on abnormal levels of free thyroxine (T_4_) and thyroid stimulating hormone (TSH), which are standard blood tests. AITD is diagnosed when these abnormal levels are found in the presence of anti-thyroid antibodies. Interestingly, it has previously been mentioned that female sex, older age and a family history of autoimmune should raise suspicion for anti-thyroid antibodies screening in children with positive ANA of unknown cause [32].

This study has strengths and limitations. First of all, due to missing onset dates for AITD cases, no association between drug therapy, disease activity, disease duration, other endocrinopathies and AITD onset could be investigated. In the current study, we observed that AITD patients had more often received TNF inhibitors than non-AITD patients. However, although a decrease in thyroid dysfunction has been reported in autoimmune disease patients treated with TNF inhibitors [40–42], we cannot draw conclusions from our study since it is unknown whether TNF inhibitor therapy was received before or after AITD onset. Another limitation of our study is that baseline comorbidities and subsequent adverse events in Pharmachild are gathered using spontaneous reporting (i.e. these have to be reported by treating physicians), which might have led to an underestimation of the actual AITD prevalence. Furthermore, it is possible that the median observation period of 5.5 years was too short for patients with young age at JIA onset to develop AITD. The exact NNS reported in this study might therefore be applicable for a follow-up period of ±5.5 years after onset of JIA only, but higher or lower afterwards. Also, the results of our study might not be generalizable to all JIA patients, since the Pharmachild registry has a selection bias towards JIA patients with a more severe disease course requiring DMARD treatment, Nevertheless, this is the first study to report independent predictors of AITD in JIA. Contrary to most of the few previous studies on AITD in JIA, we report only symptomatic AITD cases and have included patients from multiple centers around the world.

Given the AUC of our prediction model, there is room for improvement in identifying other relevant predictive factors for AITD in JIA. Further research should therefore focus on incorporating drug therapy and disease duration. As suggested previously [10], the incidence of AITD increases with time from diagnosis of JIA and therefore disease duration might be a better predictor than age at JIA onset. Another relevant predictor might be iodine intake, since it is well-described that AITD is more common in iodine-replete areas around the world [22, 26, 43, 44].

## Conclusions

To conclude, this is the first study to report independent predictors for AITD in JIA. These results provide evidence for the added value of yearly serological screening for AITD in ANA positive girls with positive family history, in order to guide a practical approach to the pediatric patient with JIA at risk of developing AITD.

## Data Availability

All relevant data are reported in the article. Additional details can be provided by the corresponding author upon reasonable request. The Pharmachild registry is registered at Clinicaltrials.gov (NCT01399281) and at the European Network of Centres for Pharmacoepidemiology and Pharmacovigilance (ENCePP; http://www.encepp.eu/encepp/viewResource.htm?id=19362).

## Abbreviations

AITD: Autoimmune thyroid disease
ANA: Anti-nuclear antibodies
b: Biological
CI: Confidence interval
cs: Conventional synthetic
DMARDs: Disease-modifying antirheumatic drugs
HLA: Human leukocyte antigen
ILAR: International League of Associations for Rheumatology
JIA: Juvenile idiopathic arthritis
NNS: Number needed to screen
NSAIDs: Nonsteroidal anti-inflammatory drugs
OR: Odds ratio
RF: Rheumatoid factor
TNF: Tumor necrosis factor
